# Development of flapping wing robot and vision-based obstacle avoidance strategy

**DOI:** 10.7717/peerj-cs.1201

**Published:** 2023-02-03

**Authors:** Heetae Park, Geunsik Bae, Inrae Kim, Seungkeun Kim, Hyondong Oh

**Affiliations:** 1 Department of Aerospace Engineering, Chungnam National University, Daejeon, Republic of Korea; 2 Department of Mechanical Engineering, Ulsan National Institute of Science and Technology, Ulsan, Republic of Korea

**Keywords:** Flapping wing micro air vehicle, Collision avoidance, Appearance variation cue, Optical flow, Indoor flight, Attitude estimation, Attitude control

## Abstract

Due to the flight characteristics such as small size, low noise, and high efficiency, studies on flapping wing robots are being actively conducted. In particular, the flapping wing robot is in the spotlight in the field of search and reconnaissance. Most of the research focuses on the development of flapping wing robots rather than autonomous flight. However, because of the unique characteristics of flapping wings, it is essential to consider the development of flapping wing robots and autonomous flight simultaneously. In this article, we describe the development of the flapping wing robot and computationally efficient vision-based obstacle avoidance algorithm suitable for the lightweight robot. We developed a 27 cm and 45 g flapping wing robot named CNUX Mini that features an X-type wing and tailed configuration to attenuate oscillation caused by flapping motion. The flight experiment showed that the robot is capable of stable flight for 1.5 min and changing its direction with a small turn radius in a slow forward flight condition. For the obstacle detection algorithm, the appearance variation cue is used with the optical flow-based algorithm to cope robustly with the motion-blurred and feature-less images obtained during flight. If the obstacle is detected during straight flight, the avoidance maneuver is conducted for a certain period, depending on the state machine logic. The proposed obstacle avoidance algorithm was validated in ground tests using a testbed. The experiment shows that the CNUX Mini performs a suitable evasive maneuver with 90.2% success rate in 50 incoming obstacle situations.

## Introduction

To this day, the flight principles of insects and birds draw researchers’ attention to the extensive possibilities of bio-inspired robots. The flight characteristics of insects and hummingbirds are especially attractive and can be alternatives to multicopters and rotorcrafts in fields such as reconnaissance missions in warfare, searching for the wounded in disasters, and toys for hobbies. Early research focused on the development of full-size or extended-size flapping wing robots. Brooks et al. (1985) created a full-sized flying replica of a pterosaur with a wingspan of 5.49 m and a weight of 18.14 kg. It could glide in the air with a ratio of 10 to 1 and could control attitude by twisting and sweeping the wings or using a spoiler on the wings. However, it was challenging to obtain thrust enough to raise or maintain the altitude just by flapping the wings. As micro-size battery and motor technology advanced, centimeter-sized flapping wing robots have been developed. Keennon & Grasmeyer (2003) proposed the Microbat, a flapping wing robot with a wingspan of 23 cm and using the control surfaces of a typical fixed-wing aircraft. It accomplished forward flight over 22 min and was found to be comparable to the performance of fixed-wing or rotary-wing aircraft at low Reynolds numbers. In 2005, the Defense Advanced Research Projects Agency (DARPA) announced the Nano Air Vehicle (NAV) program, which aims to develop a biologically inspired unmanned air vehicle capable of flying forward up to 10 m/s and hovering while maintaining a size under 7.5 cm in all dimensions. Following the NAV program, Keennon, Klingebiel & Won (2012) originated a two-winged flapping wing micro air vehicle named the Nano hummingbird. Unlike other flapping wing robots that rely on the tail and wake of the main wing to generate control, it can cover wide flight envelopes from hovering to forward flight while utilizing the propulsion of the wing as control moments. In addition, it showed that reconnaissance could be performed with a nano-sized flapping wing robot by transmitting video from the onboard camera to the ground control station. In 2014, a 3 cm flapping wing robot has been developed by using a piezo actuator and succeeded in flight with an external power source and the onboard gyroscope (Fuller et al., 2014). Beyond simply developing flapping wing robots, recent research continues to elucidate the flight principles and imitate behaviors of natural flyers. Truong, Phan & Park (2019) presented a robot based on a combination of flapping and jumping as a method that can be effectively used to overcome unspecified terrain or avoid large obstacles. The use of flapping and jumping at takeoff improved the reachable altitude by 30%, but for practical applications, it faced stabilization issues after jumping. Phan & Park (2020) noticed that the folds of the wing act as a shock absorber when the beetle strikes its wing in flight. They hypothesized that the unfolding could passively occur with the aid of the flapping forces, implemented the foldable wing with a bending joint located at the mid-span of the leading edge, and thereby the same behaviors were reproduced with a flapping wing robot during collisions. Besides, Tu et al. (2021) studied the effects of damaged wings from collisions or external factors and proposed adaptive control laws to compensate for the effects and maintain flight capability. A new study on the relation between the flapping-induced vibrations and flight stability has been published and attracts attention. Taha et al. (2020) and Karásek (2020) reported “vibrational stabilization”, a novel mechanism of insect flight that augments stability originating from body oscillations, and found that the contribution of the vibrational stabilization increases as the flapping frequency of natural flyers decreases. The authors mentioned that vibrational stabilization could be one of the design parameters in larger flapping wing robots.

Flapping wing robots can have various configurations. Firstly, according to the wing configuration, they are divided into monoplane, biplane, and tandem. It is an important parameter that determines aerodynamic efficiency and a rocking amplitude which means the movement of the body while wings are flapping. The monoplane wing is the two-winged configuration found in most insects and provides the higher thrust with the same wing size, but also generates higher rocking amplitude. The biplane and tandem wings have equally four wings but differ in the way the wing pairs are placed. When the wing pairs are placed up and down, this is called biplane or X-type configuration. The biplane wings have high power efficiency compared to the monoplane wings and small rocking amplitude with the smallest force and moment deviation during one flap cycle (Lentink, Jongerius & Bradshaw, 2010; Tay, Oudheusden & Bijl, 2014). Another classification is based on the presence or absence of a tail wing. Tailed flapping wing robots are passively stable and vibration-damped, but their flight envelope is limited to forward flight. Tailless flapping wing robots have high agility and a wide flight envelope from hovering to fast forward flight, but active stabilization is essential. As a result, control methods of flapping wing robots can be different with their configuration. In the tailed flapping wing robots, the stabilization and control are mainly *via* the tail. An biplane flapping wing robot named DelFly Explorer used the control surface of fixed-wings and demonstrated its flight ability in forward flight (De Wagter et al., 2014; Verboom et al., 2015). However, the control effectiveness of the aileron-based yaw control mechanism was poor in near hovering. On the other hand, the tailless flapping wing robots have focused on wing-based control method such as wing twist and rotation control (Karásek et al., 2018; Keennon, Klingebiel & Won, 2012; Roshanbin, Garone & Preumont, 2019), stroke plane control (Coleman et al., 2015; Nguyen & Chan, 2018; Phan et al., 2018), and flap amplitude control (Coleman et al., 2015; Zhang, Cheng & Deng, 2016), which can produce sufficient control moments. However, this configuration and control methods inevitably suffered from severe oscillations, affecting the performance of the onboard system and making it difficult to control. To actively stabilize flapping wing robots, angular velocity feedback control was carried out and verified through flight experiments in the early of the research (Fuller et al., 2014; Keennon, Klingebiel & Won, 2012). For more accurate control, attitude is also fed back into the control loop with the state estimation (Aurecianus et al., 2018; Kajak et al., 2019; Verboom et al., 2015). In addition to gyroscopes, accelerometers are used to robustly estimate attitude by filtering data (Verboom et al., 2015) or compensating *via* aerodynamic forces modeling under a quasi-steady state (Tu et al., 2018). Also, magnetometers can be used alone for pitch and yaw control, paying attention to the fact that it is not affected by flapping wings (Helbling, Fuller & Wood, 2014).

For flapping wing robots to fly autonomously in cluttered environments without collision, it is necessary to recognize and avoid obstacles quickly. Although LiDAR sensors directly provide distance information from the surrounding environment, due to strict constraints on the robot’s payload and energy, it is not applicable for small, light-weight robots. Instead, vision-based obstacle avoidance algorithms have been studied for small robots because cameras can be miniaturized but provide a wide field of view. However, since the camera does not directly provide the distance to the surrounding obstacles, an additional computational process is required to collect the surrounding environment from the image. Although various studies on vision-based obstacle detection using computer vision or deep learning algorithms have been conducted (Cao, Ren & Sun, 2022), it could be difficult to implement heavy vision algorithms or deep neural networks on a light-weight MAV. Among vision-based obstacle avoidance algorithms, optical flow-based approaches are one of the most computationally efficient algorithms with reasonable performance (Cho, Kim & Oh, 2019; Souhila & Karim, 2007). However, considering the onboard computation and sensor weight, the weight of the camera that can be mounted on the flapping wing robots should be more lighter than that used in previous studies, and it should be taken into account that the performance of the cameras decreases along with the weight. Moreover, images must be processed at a lower resolution considering real-time onboard computations for further development. In addition, mechanical vibrations generated during flight cause motion blur, which makes optical flow estimation more difficult. These challenges make it difficult for optical flow-based obstacle avoidance algorithms to be robustly applied in arbitrarily complex environments. This imposes additional challenges to optical flow estimation and these hinder the performance of the overall collision avoidance algorithm. To deal with those problems, De Croon et al. (2012b) proposed a vision-based obstacle avoidance technique using appearance variation cues to avoid obstacles in such environments with small cameras. The appearance variation cue described in detail later uses the distribution of a specific pattern in the image rather than high-frequency information such as edges, so it has robust characteristics against image noise and requires only small computation time. Although the flapping wing robot is more robust against a wall collision than the rotary robot because it does not have a high-speed rotating propeller, the flapping wing robot is more fragile due to the limited installation of an exoskeleton for protecting the robot, such as a prop guard, and is generally made of soft materials. Therefore, it can be seen that higher recall than precision is more essential for long-term operation of the flapping wing robot in case of collision. In this article, to robustly detect obstacles, we propose the sequential two-phase detection structure that first detects obstacles in front of robots and seeks horizontal obstacles next. Even if the first obstacle detector misses the obstacle, the next detector can detect the obstacle, increasing the detection sensitivity; this improves the sensitivity of the detection algorithm compared with De Croon et al. (2012b) and makes it easier to tune the parameters (*e.g*., threshold). More specifically, in this article, the optical flow-based algorithm is used to detect left and right obstacles, and the appearance variation cue is set to perform obstacle avoidance for the front obstacles.

In this work, we introduce the CNUX Mini developed for autonomous flight based on a vision system. Compared to our previous robot (CNUX IV), it can accurately change its direction with a novel wing-based heading control mechanism and has an extended flight envelope from near hovering to slow forward flight. It also has a unique feature in that the thrust-to-wingspan ratio is higher than that of other flapping wing robots with similar wingspans. This characteristic implies that the robot can carry many payloads even with a slightly smaller size, which is advantageous for reconnaissance and camouflage. Furthermore, we combine the optical flow-based algorithm and appearance variation cues-based algorithm to identify incoming obstacles during flight. And using these capabilities of object detection, we present turning logic for avoiding obstacles. The proposed algorithm effectively copes with frontal obstacles by utilizing appearance variation cues and horizontal obstacles using optical flow.

The article is organized as follows. The first section briefly looks over our previous research, presents the mechanical design of the flapping mechanism to drive the wing, and explains the wing-based heading control methodology. The second section describes filtering methods for sensor data contaminated by flapping motion and shows algorithms to estimate and control attitude. Then, the subsequent section proposes an obstacle detection and avoidance algorithm using vision systems. The last section evaluates the performance of the flapping wing robot and obstacle algorithm through experiments and discusses the results.

## Development of cnux mini

### Overview

**Figure 1:**
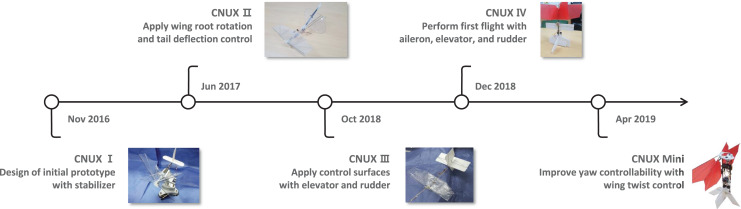
The CNUX flapping wing robot development history.

**Figure 2:**
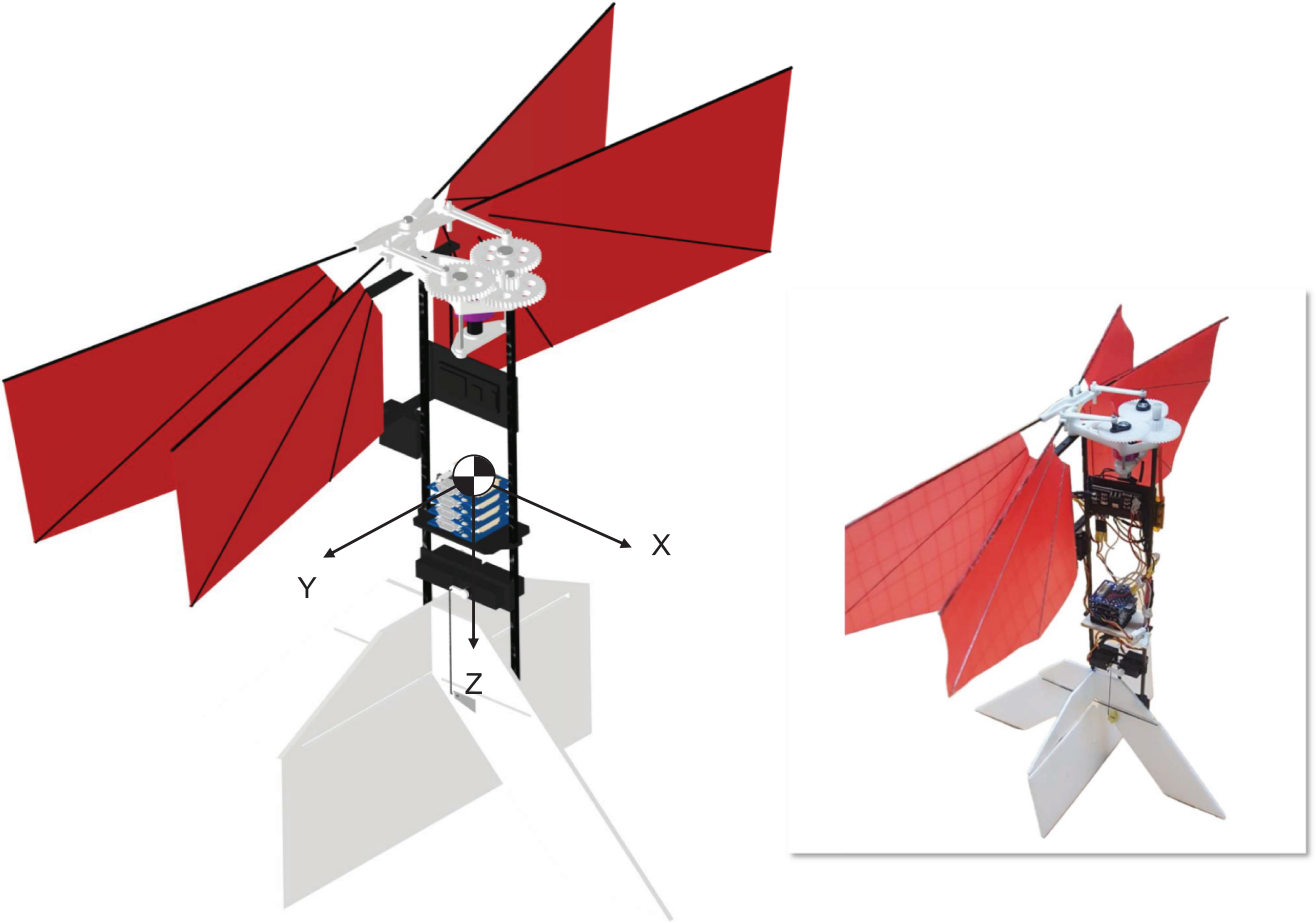
3D CAD design and the CNUX Mini flapping wing robot.

**Figure 3:**
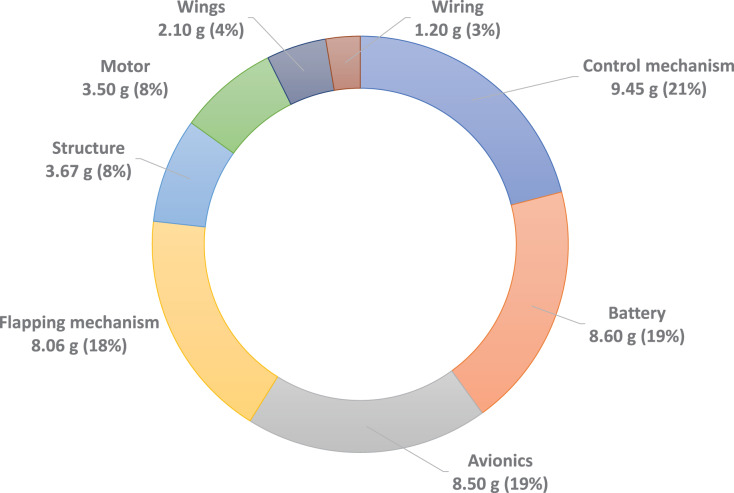
The CNUX Mini weight breakdown.

The CNUX is the flapping wing robot has been developed at Chungnam National University since 2016. Figure 1 shows the CNUX developed by year and their features. The CNUX I to III aimed at the development and application of new control mechanisms, while the CNUX IV succeeded in its first free flight. As shown in Fig. 2, the CNUX Mini is a flapping wing robot with a wingspan of 27 cm and a weight of 45 grams, which features two pairs of wings equivalent to an X-type configuration and has a high thrust-to-wing span ratio. The X-type configuration makes the interaction by the clap and fling effect during stroke reversal and attenuates the body oscillations because the top and bottom wings have opposite phases. In order to create large thrust, it is common to increase the flap amplitude and lower the flapping frequency as much as possible. Since there are four wings on the stroke plane, the robot was developed in the direction of increasing the flapping frequency to create large thrust. Figure 3 and Table 1 show the weight breakdown and specifications of the CNUX Mini. Although the control mechanism accounts for 21% of the total weight due to the tail, it can provide damping force and can stabilize the robot without active control.

**Table 1:** The CNUX mini specifications.

Parameters	Value
Total mass	45 g
Wing span	27 cm
Mean chord	6.3 cm
Flap amplitude	40 deg
Flapping frequency	25 Hz

### Flapping mechanism

**Figure 4:**
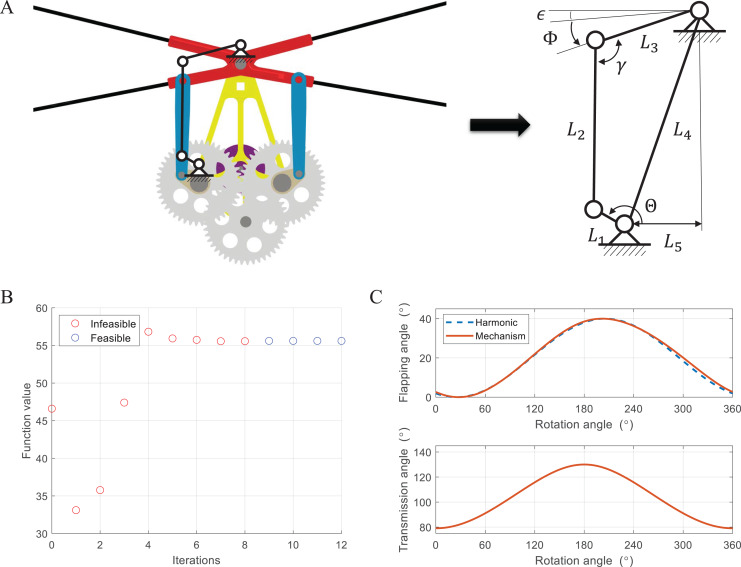
Conceptual design and kinematic diagram of the flapping mechanism (A), cost and feasibility during iteration (B), and optimized flapping angle and transmission angle (C).

The flapping motion can be implemented through a power source and a flapping mechanism. Power is generated by a brushless DC motor and converted to the appropriate torque and rotational speed *via* a gearbox. There are various types of flapping mechanisms such as linkage-based (Jeon et al., 2017; Roshanbin et al., 2017; Tijmons, Karásek & Croon, 2018), string-based (Gong et al., 2019; Phan et al., 2020), and gear-based ones (Lee & Kim, 2019; Preumont et al., 2021). At the beginning of research about flapping wing robots, the linkage-based mechanism was mainly used due to its simple structure and decent performance. However, it can not generate large flap amplitude only using one stage and suffers from big inertia forces during the stroke, which degrades efficiency and often requires repair. To reduce the size and increase efficiency, it was changed to string-based or gear-based mechanisms, which have fewer moving parts and easy to produce large flap amplitude. They also face several issues with the mechanical power transmission and the symmetry of both wings. Since our flapping wing robot has four wings, a relatively small flap amplitude is suitable as mentioned earlier. Therefore, we exploit the symmetric four-bar linkage-based mechanism. Figure 4A illustrates the conceptual design and kinematic diagram. The four-bar linkage consists of a crank as an input, a rocker as an output, and a coupler as an intermediate link between them. The analytic solution can be described as:

$\Phi ({L},\Theta ) = 2\arctan \left(\displaystyle{{{L_1}\sin (\Theta ) - {L_2}\sin(\gamma )} \over {{L_1}\cos (\Theta ) + {L_3} - {L_4} - {L_2}\cos (\gamma )}}\right) - \displaystyle{\pi \over 2} - \arcsin \left(\displaystyle{{{L_5}} \over {{L_4}}}\right) - \varepsilon$

$\gamma ({L}) = \arccos \left(\displaystyle{{L_2^2 + L_3^2 - {S^2}} \over {2{L_2}{L_3}}}\right)$

$S({L},\Theta ) = \sqrt {L_1^2 + L_4^2 - 2{L_1}{L_4}\cos (\Theta )}$

where ${L_1}$ is the length of the crank, ${L_2}$ is the length of the coupler, ${L_3}$ is the length of the rocker, ${L_4}$ is the distance between two pivot points, and ${L_5}$ is the horizontal distance between two pivot points. $\Theta$ is the rotation angle of the main gear, $\Phi$ is the the flapping angle, $\gamma$ is the transmission angle defined as the angle between the coupler and the rocker, and ${\varepsilon }$ is the clearance angle meaning the minimum angle between the top and bottom wings for safety.

The motion of four-bar linkages can vary in accordance with the combination of link length. To ensure reciprocating motion and achieve the desired performance, we carried out the optimization of link length. The optimization problem can be expressed in Eq. (1). The cost function is defined as the sum of error between the transmission angle and $\pi /2$, which leads to producing smooth motion. The first and second constraints assure that the desired flap amplitude and minimum angle are met. The third constraint guarantees that the minimum and maximum transmission angle is within given bounds. The fourth constraint makes the four-bar linkage to be the crank rocker mechanism according to the Grashof criterion (Grashof, 1883). Here, we set that ${\Phi _{desired}}$ is 40°, $\varepsilon$ is 3.5°, ${\gamma _{max}}$ is 140° and ${\gamma _{min}}$ is 40°. $s$ is the length of the shortest link, $l$ is the length of the longest link, and $p$ and $q$ are the lengths of remaining links.

(1) $$\mathop {{\rm minimize}}\limits_{\bf L} \quad J({\bf L}) = \sum\limits_{i = 1}^n {{{\left({\gamma _i}({\bf L}) - \displaystyle{\pi \over 2}\right)}^2}}$$

$\matrix{ {{\rm subjected\; to}\quad } & {{\Phi _{max}} - {\Phi _{min}} = {\Phi _{desired}},} \cr {} & {{\Phi _{min}} = 0,} \cr {} & {{\gamma _{min}} < \gamma < {\gamma _{max}},} \cr {} & {s + l < p + q} \cr }$

The optimization was sequentially conducted by GA (Genetic Algorithm) and fmincon solver in MATLAB. GA first finds the near-global minimum and then, fmincon solver determines the global minimum based on the results of GA. Figure 4B shows the cost function value as GA optimization progresses. GA found a feasible solution after eight iterations and successfully optimized it. Figure 4C shows the optimized flapping angle and transmission angle. To verify the results, the flapping angle was compared with a harmonic function of the same amplitude, which has smooth characteristics in terms of velocity and acceleration. The trajectory is overall similar during one cycle, and it was confirmed that the closer the transmission angle and $\pi /2$ on the four-bar linkage, the more harmonic the motion occurs. Finally, the flapping mechanism is fabricated by 3D printing using Polyamide-6 material with excellent wear resistance and mechanical strength, and gears use off-the-shelf products made of POM (Polyoxymethylene), a type of engineering plastic.

### Wing

**Figure 5:**
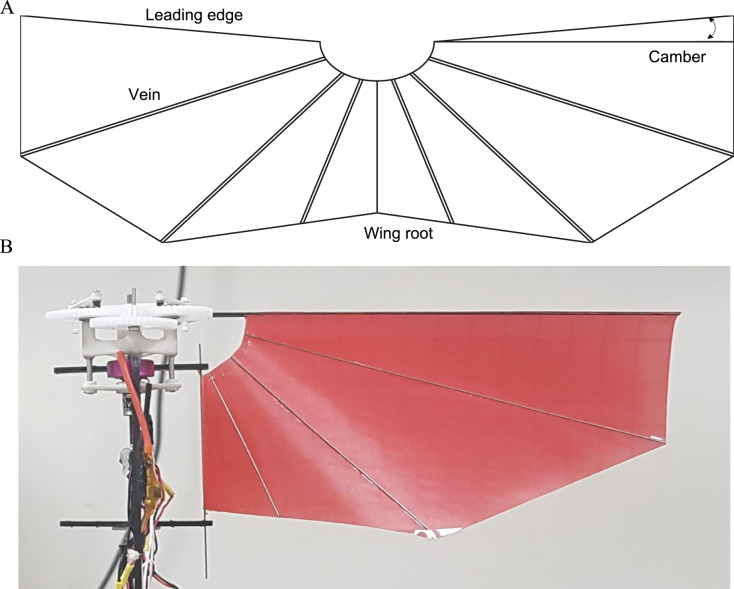
Wing configuration (A) and deformation after wing equipment (B).

The wing basically consists of the wing membrane, the leading edge, the trailing edge, and the veins as shown in Fig. 5A. Polyester film with 0.07 mm thickness is used for the wing membrane, which is durable enough to withstand the force generated during the stroke and lightweight enough to minimize inertial forces. The leading edge and wing root are composed of carbon fiber rod 1.0 and 0.5 mm, respectively. They are fully attached to the wing instead of using the sleeve to rotate passively around an axis. This corresponds to limiting the degree of freedom of the wing and reducing the uncertainty between the wings. Vein is also made with 0.5 mm carbon fiber rod and its configuration is of a diagonal pattern, which is common layout in flapping wing robots. Since the wing has an extra area by the camber angle, the wing membrane becomes loose when mounted on the leading edge and wing root as shown in Fig. 5B. An appropriate camber angle is known to reduce drag and increase efficiency (Nan et al., 2017; Yoon et al., 2019) and was set to 5° in our robot. The wing was fabricated by cut and glue method.

### Control mechanism

In our previous work (CNUX IV), the control surfaces of the fixed-wing such as ailerons and elevators was used for stabilization and control. Longitudinal and lateral stability could be sufficiently augmented through a tail providing additional damping force, but maneuverability was lacking in all axes. Control effectiveness problem arose in operating conditions that are low-speed flight or near hovering, since the wake caused by flapping only affects the generation of control moments. Especially, the heading change was possible only if the forward speed is sufficient. To deal with this problem, we enlarged the deflection area of the rudder and elevator similar to an all-moving tail, and a wing-based control mechanism was applied for yaw control.

**Figure 6:**
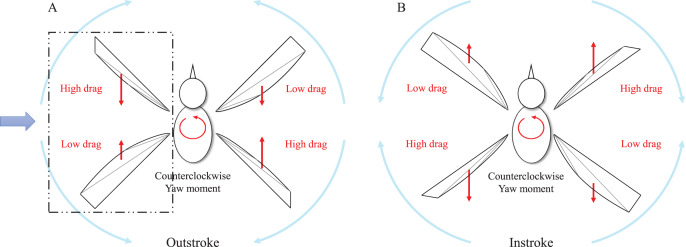
Schematic view of counterclockwise yaw control moment generation during one flap cycle. Force distributions in the outstroke (A) and in the instroke (B).

**Figure 7:**
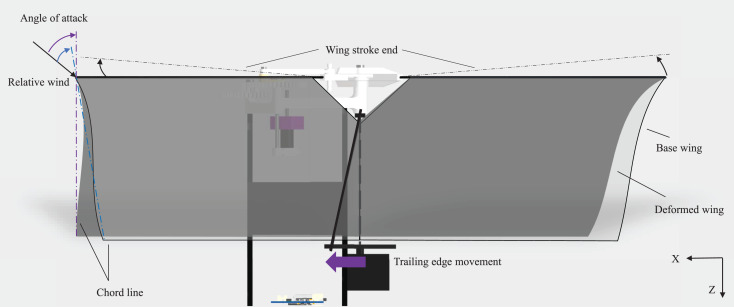
Side view of counterclockwise yaw control moment generation during the outstroke.

The wing-based control was proposed in Keennon, Klingebiel & Won (2012) and has been mainly used by lots of flapping wing robots with a pair of wings. When the wing-based control is applied directly to our flapping wing robot with two pairs of wings, the aerodynamic forces and moments should be verified during the entire stroke. One cycle of flapping consists of the outstroke and instroke as shown in Figs. 6A and 6B. The outstroke is referred to the phase in which the fore and aft wings move away from the horizontal line of the body. The instroke is referred to the phase in which the fore and aft wings come together based on the horizontal line of the body. The basic principle of the wing-based control mechanism is to adjust the angle of attack, which means the angle between the chord line and the relative wind. Depending on the angle of attack, the magnitude of thrust and drag is changed. To create a yaw control moment, we assume that the left trailing edge moves forward and the right trailing edge moves backward. Figure 7 illustrates the dotted rectangular area as viewed from the blue arrow in Fig. 6. When the trailing edge of the left-wing pairs moves forward as shown by the purple arrow in the Fig. 7 (dotted line to solid line), the shape of the wing changes from the base wing to the deformed wing. This results in the fore wing with a relatively high angle of attack (purple angle) and the aft wing with a relatively low angle of attack (blue angle). In other words, the fore wing has a relatively high drag distribution and the aft wing has a relatively low drag distribution during the outstroke. Similarly, in the right-wing pair, the opposite result is obtained because the trailing edge moves backward. Consequently, the moment in the same direction is continuously generated in the outstroke and the instroke.

## Estimation and control

### Data filtering

**Figure 8:**
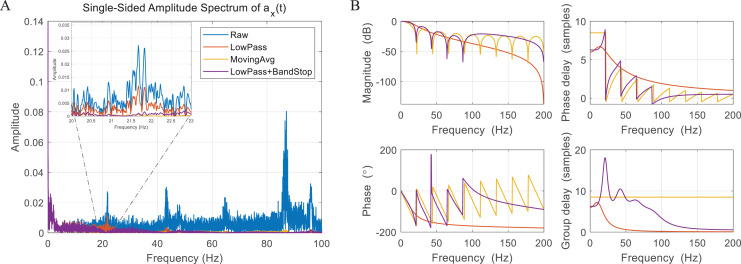
Amplitude spectrum of accelerometer data (A) and filter performance analysis (B).

The flapping wing robot is equipped with an Micro Controller Unit (MCU) based on ARM Cortex M0+ and a nine-axis Inertial Measurement Unit (IMU), which consists of a gyroscope measuring angular velocity, an accelerometer measuring linear and gravity acceleration, and a magnetometer measuring the change in the magnetic field. The flapping motion causes the body to oscillate periodically and generates additional high-frequency noise. The oscillation seriously contaminates sensor data, especially in the gyroscope and accelerometer. Figure 8A shows an amplitude spectrum of acceleration about the x-axis on the body. In the raw data, one can identify that the data includes a signal in a band corresponding to an integer multiple of the flapping frequency 21.8 Hz, that is, colored noise. We compared three types of filtering methods to attenuate the colored noise. The first one is a second-order Butterworth lowpass filter with a cutoff frequency of 15 Hz, and the filter order and cutoff frequency were determined in consideration of time delay and attenuation level. The second one is the moving average filter, which is a technique using equal weights for samples up to window size and was successfully applied to accelerometers filtering (Verboom et al., 2015). Here, we used the window size of 18 because the signal-to-noise ratio improves as the window size becomes similar to the wingbeat. The third one is a combination of a Butterworth lowpass filter and a bandstop filter to effectively attenuate the colored noise and minimize time delay. The bandstop filter directly covers the colored noise in bands of once, twice, three times, and four times the flapping frequency, and the lowpass filter handles the high-frequency noise as the first-order filter with a wide passband. The amplitude spectrum of filtered data and filter frequency response are shown in Figs. 8A and 8B. It can be seen that the cutoff frequency and the flapping frequency are close in the lowpass filter, so the colored noise cannot be effectively blocked. As shown in the magnitude plot, the moving average filter minimizes the effect of colored and white noise, but it also filters slow bands which contain the real body motion that needs to be captured and fed back into the controller. In addition, the moving average filter is a type of non-recursive FIR (Finite Impulse Response) filter, so it is more vulnerable to phase delay and group delay than other filters. The combination of the lowpass filter and the bandstop filter shows the best performance in terms of signal preservation, noise block, and phase delay because the signal passes as fully as possible in the slow band and the colored noise is strongly attenuated. Therefore, we determined to use the lowpass filter and the bandstop filter for filtering gyroscope and accelerometer measurements.

### Single sensor-based estimation

After the data is filtered, the measurements of the gyroscope and accelerometer include the bias and noise, which can be modeled as Eqs. (2) and (3). It is assumed that the bias is slowly time-varying, and the noise is close to white *via* data filtering.

(2) $${{\rm \omega }_m} = {{\rm \omega }_t} + {{\rm \omega }_b} + {\rm \eta }$$

(3) $${{\bf a}_m} = {{\bf a}_l} - {{\bf a}_g} + {{\bf a}_b} + {\bf \xi }$$

where ${\rm \omega } = {\left[ {p,\,q,\,r} \right]^T}$ is the gyroscope measurements vector, ${\bf a} = {[{a_x},\;{a_y},\;{a_z}]^T}$ is the accelerometer measurements vector, subscript $m$ denotes the measurements, $t$ represents the true value of sensors, $b$ means the bias of sensors, $l$ denotes the linear acceleration, $g$ is the gravity acceleration in the body frame, and ${\rm \eta }$ and ${\bf \xi }$ are white noise of the sensors.

The kinematics about Euler rates and angular velocities are expressed as Eq. (4) (Madyastha et al., 2011). By integrating the differential equation, the gyroscope-based attitude can be calculated. The gyroscope can estimate the attitude, but the bias consistently induces integration error.

(4) $$\mathop {\widehat {\bf{x}}}\limits^.  = f({\widehat {\bf{x}}_{\bf{k}}}) = \left[ \matrix{
  {f_1}(\widehat {\bf{x}}) \hfill \cr 
  {f_2}(\widehat {\bf{x}}) \hfill \cr 
  {f_3}(\widehat {\bf{x}}) \hfill \cr}  \right] = \left[ \matrix{
  p + q\sin {{\hat x}_1}\tan {{\hat x}_2} + r\cos {{\hat x}_1}\tan {{\hat x}_2} \hfill \cr 
  {\mkern 1mu} {\mkern 1mu} {\mkern 1mu} {\mkern 1mu} {\mkern 1mu} {\mkern 1mu} {\mkern 1mu} {\mkern 1mu} {\mkern 1mu} {\mkern 1mu} {\mkern 1mu} {\mkern 1mu} {\mkern 1mu} {\mkern 1mu} {\mkern 1mu} {\mkern 1mu} {\mkern 1mu} {\mkern 1mu} {\mkern 1mu} {\mkern 1mu} {\mkern 1mu} {\mkern 1mu} {\mkern 1mu} {\mkern 1mu} {\mkern 1mu} {\mkern 1mu} {\mkern 1mu} {\mkern 1mu} {\mkern 1mu} {\mkern 1mu} {\mkern 1mu} {\mkern 1mu} {\mkern 1mu} {\mkern 1mu} {\mkern 1mu} {\mkern 1mu} {\mkern 1mu} q\cos {{\hat x}_1} - r\sin {{\hat x}_1} \hfill \cr 
  {\mkern 1mu} {\mkern 1mu} {\mkern 1mu} {\mkern 1mu} {\mkern 1mu} {\mkern 1mu} {\mkern 1mu} {\mkern 1mu} q\sin {{\hat x}_1}\sec {{\hat x}_2} + r\cos {{\hat x}_1}\sec {{\hat x}_2} \hfill \cr}  \right]$$

where ${\hat {\bf x}} = {[\hat \phi ,\;\hat \theta ,\;\hat \psi ]^T}$ refers to the gyroscope-based attitude vector, and ${x_i}$ means i-th state variable. Also, the accelerometer-based attitude ${\bf z} = {[{\phi _m},\;{\theta _m}]^T}$ can be estimated under conditions of zero linear acceleration ${{\bf a}_l} = {\bf 0}$, such as the hovering and flight at constant velocity. The relation between accelerometer measurements and the attitude is given by Eq. (5) (Euston et al., 2008). The accelerometers provide time-independent absolute measurements, but are very sensitive to high-frequency motion.

(5) $${\bf z} = [\arctan 2( - {a_y}, - {a_z}),\quad \arctan 2{({a_x},\sqrt {a_y^2 + a_z^2)} ]^T}{\rm }$$

### Complementary filter

**Figure 9:**
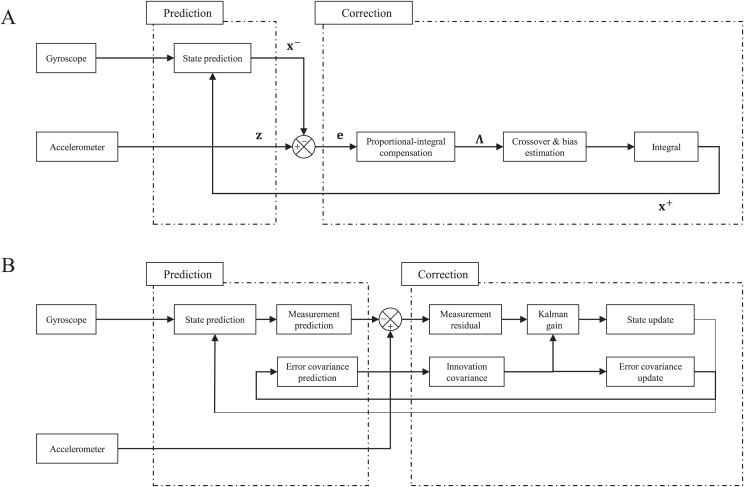
Block diagram of complementary filter (A) and Kalman filter (B).

The complementary filter is based on the fact that the gyroscope has good response and sensitivity in the high-frequency band, whereas the accelerometer does in the low-frequency band. Figure 9A depicts the block diagram of the complementary filter, which is composed of the prediction and correction stages. In the prediction, the Euler rates are calculated with the gyroscope measurements and the posteriori state at the previous step using Eq. (4). Then, the priori state is estimated as in Eq. (6) *via* Euler’s approximation considering only the first-order term.

(6) $$\widehat {\bf{x}}_{k + 1}^ -  = \widehat {\bf{x}}_k^ +  + \mathop {\widehat {\bf{x}}}\limits^. \Delta t + {{\mathop {\widehat {\bf{x}}}\limits^{..} \Delta {t^2}} \over 2} + ... \approx \widehat {\bf{x}}_k^ +  + \mathop {\widehat {\bf{x}}}\limits^. \Delta t$$

where $\Delta t$ is the sampling time, and ${{\hat {\bf x}}^ - }$ and ${{\hat {\bf x}}^ + }$ denote the priori and posteriori state, respectively. In the correction step, the predicted state variables are updated using the accelerometer-based attitude. The compensation values $\Lambda$ are calculated using the error vector ${\bf e} = {\bf z} - {{\hat {\bf x}}^ - }$ through the Proportional-Integral (PI) structure proposed in Euston et al. (2008). In the PI structure, the posterior state is equivalent to the sum of the highpass filtered gyroscope-based attitude and the lowpass filtered accelerometer-based attitude. The time derivative of the bias estimate in the inertial frame can be indicated as an integral term and continues to accumulate.

$$\eqalign{
  & \Lambda  = {K_p}{\bf{e}} + {K_i}\int_{k\Delta t}^{k\Delta t + 1} {\bf{e}} (\tau )d\tau   \cr 
  & \dot{\hat{\omega}}_{b^{G}} =  - {K_i}\int_{k\Delta t}^{k\Delta t + 1} {\bf{e}} (\tau )d\tau  \cr} $$

where ${\omega ^G}$ is the bias in the inertial frame, the ${K_p}$ is the proportional gain that determines the crossover band of two sensors, and ${K_i}$ is the integral gain related to the frequency band of the bias. Finally, the posteriori state and bias estimate are yielded as:

$$\eqalign{
  & {\bf{\hat {x}}}_{{k + 1}^ {+}}  = {\bf{\hat {x}}}_{{k + 1}^ {-}}  + \Lambda \Delta t  \cr 
  & \hat {\omega} _{{b,k + 1}^{G}} \approx \hat {\omega} _{{b,k}^{G}} + {\dot {\hat {\omega}}} _{b^{G}}\Delta t \cr} $$

### Extended Kalman filter

The Kalman filter is known as an optimal linear unbiased estimator, which can deal with systems including noise, and the block diagram is represented in Fig. 9B. The system we want to handle can be defined as a continuous nonlinear process model and discrete linear measurement model as follows.

$\eqalign{& {\dot {\bf x}}(t) = f({\bf x}(t)) + \Gamma {\bf w}(t) \cr & {{\bf z}_k} = H{{\bf x}_k} + {{\bf v}_k}}$

where $f({\bf x}(t))$ is the nonlinear process model, $\Gamma$ is the process noise scaling matrix, ${\bf w}(t)$ is the Gaussian process noise with ${\rm {\cal N}}(0,Q)$, and ${{\bf v}_k}$ is the Gaussian measurement noise with ${\rm {\cal N}}(0,R)$. It is assumed that the process noise and measurement noise are uncorrelated to each other as Eqs. (7) and (8) (Madyastha et al., 2011).

(7) $${\rm {\mathbb E}}(w(t)w{(\tau )^T}) = \left\{ \matrix{Q,\quad t = \tau \cr 0,\quad t \ne \tau} \right.$$

(8) $${\rm {\mathbb E}}({v_i}v_j^T) = \left\{ \matrix{R,\quad i = j \cr 0,\quad i \ne j } \right.$$

where ${\rm {\mathbb E}}$ denotes expectation. The continuous process model can be handled to the discrete model by *Euler’s approximation* of Eq. (6). The state transition and process noise covariance matrix in the discretized model are defined as (Lewis, Xie & Popa, 2008; Madyastha et al., 2011):

$\eqalign{& {A_{s,k}} = I + {A_k} + \displaystyle{{A_k^2\Delta{t^2}} \over {2!}} + ... \approx I + {A_k}\Delta t \cr & {Q_{s,k}} = {\Gamma _k}Q\Gamma _k^T\Delta t + \displaystyle{{({A_k}{\Gamma _k}Q\Gamma _k^T + {\Gamma _k}Q\Gamma _k^TA_k^T)\Delta{t^2}} \over {2!}} + ... \approx {\Gamma _k}Q\Gamma _k^T\Delta t}$

where subscript $s$ denotes discretization, *A* is the state transition matrix obtained by the Jacobian of $f({\bf x}(t))$, and *Q* is the process noise matrix. In the prediction of the Kalman filter, the priori state ${\bf x}_{k + 1}^ -$ and the error covariance $P_{k + 1}^ -$ are calculated by the Eqs. (6) and (9). This corresponds to the step of predicting the attitude of the next step with the measurements of the gyroscope.

(9) $$P_{k + 1}^ - = {A_{s,k}}P_k^ + A_{s,k}^T + {Q_{s,k}}$$

The correction of the Kalman filter updates values of the prediction step with measurements. First, the residual ${\bf \nu }$ is computed by the errors between the accelerometer-based attitude ${\bf z}$ in Eq. (5) and predicted measurements of the process model in Eq. (6).

${{\bf \nu }_{k + 1}} = {{\bf z}_{k + 1}} - H\hat x_{k + 1}^ -$

The residual indicates how far the predicted value is from the measured value and is combined with the Kalman gain to determine the amount of update. The Kalman gain is obtained by minimizing a norm of the estimation error and is given by

${K_{k + 1}} = P_{k + 1}^ - H_{k + 1}^T{({H_{k + 1}}P_{k + 1}^ - H_{k + 1}^T + R)^{ - 1}}$

Finally, the posteriori state and error covariance can be represented as Eqs. (10) and (11). Detailed derivation can be found in Lewis, Xie & Popa (2008).

(10) $${\hat {\bf x}}_{k + 1}^ + = {\hat {\bf x}}_{k + 1}^ - + {K_{k + 1}}{\nu _{k + 1}}$$

(11) $$P_{k + 1}^ + = (I - {K_{k + 1}}{H_{k + 1}})P_{k + 1}^ -$$

### Controller

**Figure 10:**
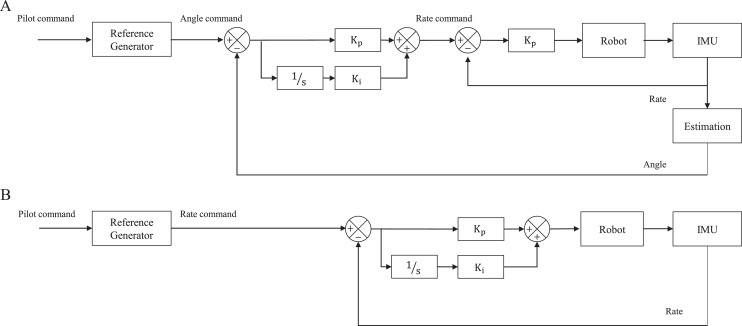
Controller architecture of attitude (roll and pitch) (A) and heading (yaw) (B).

Considering the development objective of our flapping wing robot, we aim to accurately control our roll and pitch angle, whereas yaw control focuses on changing direction from the current position when detecting obstacles, *i.e*., controlling the yaw rate. The control architecture is configured separately for each axis as shown in Fig. 10. Pilot commands *via* a transmitter or Ground Control Station (GCS) are passed to the reference generator which is a Butterworth second-order low pass filter and thereby makes the pilot commands smooth (Kajak et al., 2019). To quickly stabilize the robot’s inherent instability, roll and pitch controllers were configured *via* a cascaded architecture composed of an outer loop and an inner loop as shown in Fig. 10A. In the outer loop, the Proportional-Integral (PI) controller is configured for the attitude error, and the output becomes the angular velocity command of the inner loop. The inner loop includes only Proportional (P) controller and its output is delivered to control surfaces. Yaw is controlled *via* the PI controller as shown in Figs. 10B to cover the Flapping Counter Torque (FCT), which is produced in the opposite direction when turning and is proportional to the square of the turning speed and causes a steady-state error.

## Obstacle detection and avoidance

### Horizontal obstacle detection algorithm

**Figure 11:**
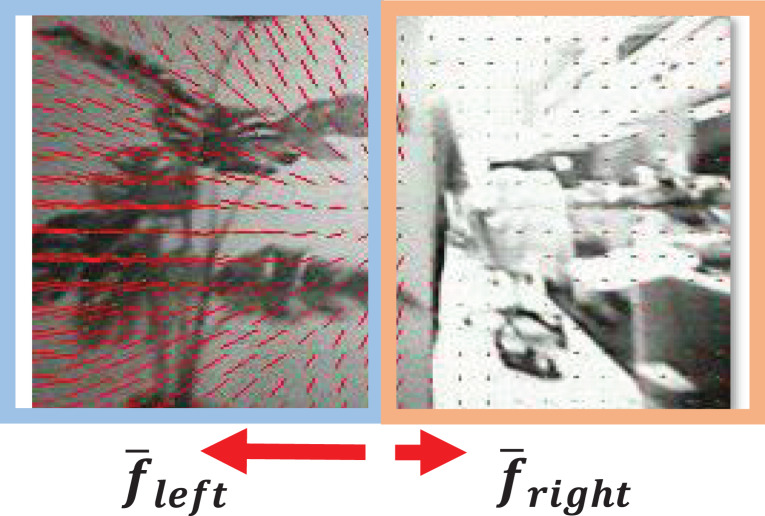
An exemplary optical flow estimation result with Horn–Schunck algorithm in the presence of a close obstacle.

The optical flow is a technique for estimating image displacements between sequences of images. The amount of change of pixel coordinates in the image can be used to obtain motion information from sequential images. In particular, when the camera moves with a fixed speed in a static obstacle environment, a nearby object in the image moves more than a distant object, which generates a large optical flow. Thus a large optical flow can be used to recognize the proximity of the obstacle. Also, if either side of the optical flow is large, it is possible to determine the direction to avoid obstacles in the direction in which the optical flow occurs low. Figure 11 shows how the optical flow is estimated when the left obstacle is close.

In this article, the optical flow is estimated by the Horn–Schunck method (Horn & Schunck, 1981), which has good computational efficiency with reasonable accuracy. Since this method does not use feature detection that requires high-resolution detail of the image, it is suitable for low-resolution cameras. In addition, if a fast operation is required, the image can be downscaled so that the calculation can be completed at the desired time. Although the accuracy of estimated optical flow could decrease using a downscaled image, it is more robust to image noise such as motion blur. The estimated optical flow is divided into a left half-plane and a right half-plane based on the center line of the image. If the difference between the sum of the optical flow at the left side ( ${\bar f_{left}}$) and the right side ( ${\bar f_{right}}$) is greater than the threshold ${\theta _{OF}}$, then we assume that there is an obstacle to be avoided with a high probability in the horizontal direction. The sum of the optical flow is calculated using the following equations.

$\eqalign{& {{\bar f}_{left}} = \sum\limits_{i \in left} {\sqrt {u_i^2 + v_i^2} } , \cr & {{\bar f}_{right}} = \sum\limits_{i \in right} {\sqrt {u_i^2 + v_i^2} } ,}$

where $({u_i},{v_i})$ means the value of the optical flow vector estimated at the ${i^{th}}$ pixel of the image.

Many existing studies (Cho, Kim & Oh, 2019; Muratet et al., 2005; Souhila & Karim, 2007) that utilize optical flows to avoid horizontal obstacles used the flow difference between the left and right half-plane to calculate a yaw rate command, $\dot \psi$, with a predefined gain, which is called the horizontal balance strategy. However, these studies were conducted on rotary-wing aircraft with relatively stable yaw control capability. Since flapping wing robots have weak yaw control capability due to their structure, frequent changes in the yaw rate command can cause flight instability due to a delayed response of the flapping wing robot. Thus, we only use the difference of optical flows as a criterion to identify the obstacle and use the simple control strategy for obstacle avoidance, *i.e*., the obstacle avoidance algorithm was implemented separately from the obstacle detection algorithm. Such a modular design facilitates the identification of problems when the aircraft collides with an obstacle due to an error, thereby improving overall performance.

### Frontal object detection algorithm

The method to estimate obstacle proximity based on appearance variation cue was first proposed by De Croon et al. (2012a) and is based on the following principle. When a camera is approaching an object, other objects are moving out of view, and only a single object fills the field of view. In general, a single object usually has fewer colors and textures than many different objects, in other words, color and texture variation is small. Thus, as the collision approaches, color and texture variations decrease, which can be used for obstacle recognition.

The optical flow-based algorithm is to identify an incoming obstacle during low-speed flight or when the difference of optical flows at the right and left half-planes is small (*i.e*., frontal obstacle case). Besides, in a monotonous environment like a hallway, it is difficult to estimate the optical flow accurately. Therefore, to perform obstacle avoidance in these harsh situations, the appearance variation cue algorithm was used as a front obstacle detector that can capture possible missing obstacles by the optical flow-based algorithm.

**Figure 12:**
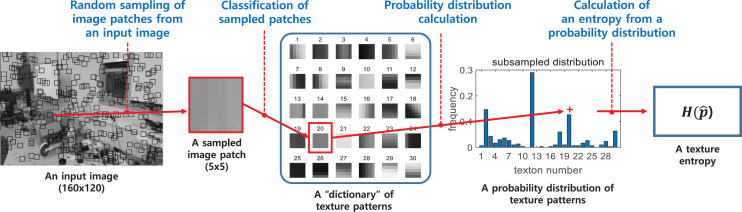
The calculation process of a texture entropy (De Croon et al., 2012b).

**Figure 13:**
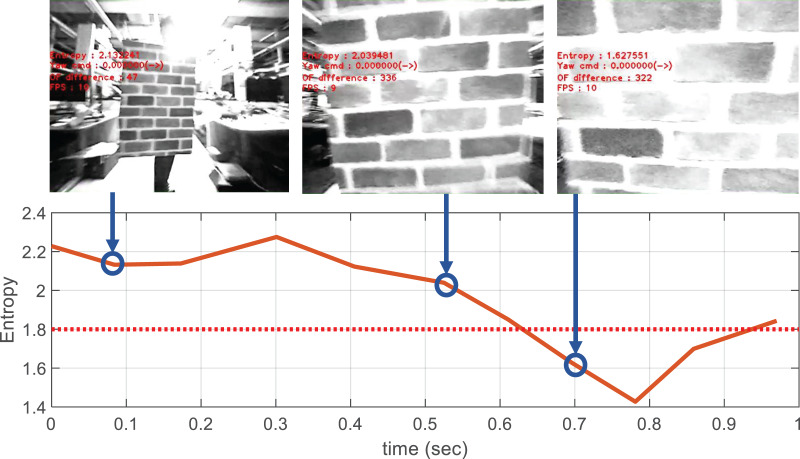
Decrease in texture entropy when approaching obstacles.

The visual appearance of an image is defined by the *texton* method (De Croon et al., 2012a), which classifies small patches within the image as a number of predefined texture patterns. It is possible to measure how many different textures the image contains based on the distribution of classified image patches. The appearance variation is calculated as the entropy of the obtained texture distribution, where the calculation process is shown in Fig. 12. Unlike optical flow, the appearance variation cue is robust against image noise because it can obtain information about the distance to an obstacle from a single image. The texture entropy tends to decrease as it approaches the obstacle, and when it falls below a specific threshold value, ${\theta _H}$, we consider that there is an obstacle with a risk of collision (De Croon et al., 2012a). The threshold value is obtained through experiments in different environments. Figure 13 shows the change in texture entropy as the obstacle gets closer.

### The control strategy for obstacle avoidance

In order to increase the success rate of obstacle avoidance of the robot, it must respond immediately to incoming visual input. Therefore, for fast obstacle avoidance, a method using simple left/right turn actions was used instead of a complex trajectory planning method that requires a long computation time. We assumed the obstacle can be avoided by changing the yaw rate command with a constant forward flight speed.

**Figure 14:**
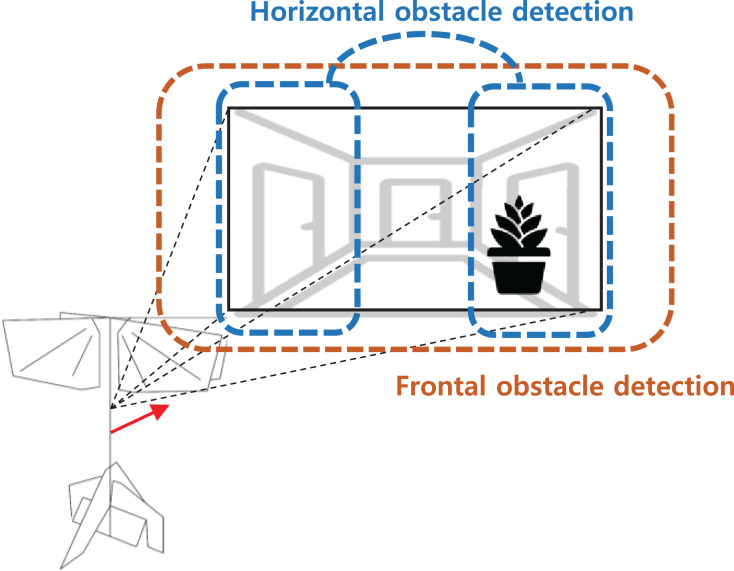
Obstacle search regions for each detectors.

**Figure 15:**
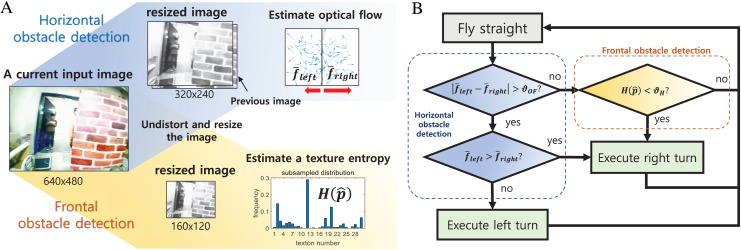
The obstacle detection (A) and avoidance algorithm (B).

The overall obstacle detection and avoidance algorithm proceed as follows. First, the distortion of an input image is corrected using intrinsic camera parameters. This allows the optical flow at the image edge to be estimated more accurately. Next, nearby obstacles are identified using both optical flow and appearance variation cues. Note that both operations are performed on the gray-scaled image. Although appearance variation cues can be defined using the color information for better performance, we used the gray-scaled image for computational and memory efficiency. Figure 14 shows regions that are covered by each detector. If there are no obstacles, the robot will keep forward flight. If a nearby obstacle is detected by the optical flow-based method, turn direction is determined by comparing the amount of optical flow between the left and right half-plane. The direction of rotation is determined by the direction in which the smaller optical flow occurs. The rotation command is maintained for a certain period, and when the rotation is finished, the robot moves forward again. For the frontal obstacle detected by the appearance variation cue algorithm, similar to the horizontal obstacle, the robot executes a right turn for a certain period when an obstacle is detected. The overall obstacle avoidance logic is shown in Fig. 15.

## Results and discussion

### Thrust measurement

**Figure 16:**
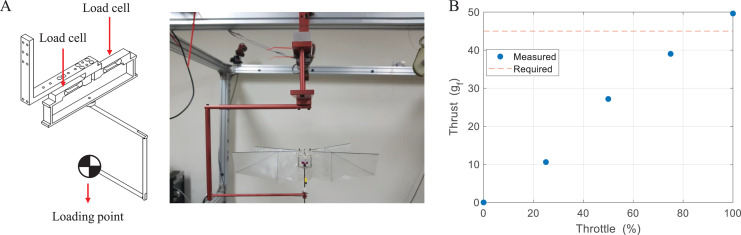
Experimental setup (A) and thrust measurement result (B).

Figure 16A shows an experimental setup that is composed of two load cells and can measure forces and moments on one axis. Thrust is measured by adding the outputs of two load cells and moment is measured by subtracting the outputs of two load cells, and the system can sample signals up to 5,000 Hz (Park et al., 2018). Thrust was measured for 5 s depending on the throttle and averaged value was calculated by the numerical integration. To accurately measure the aerodynamic force, the experiment was performed in the state where the center of gravity of the robot was fixed at the loading point. Thrust was measured equivalent to the weight of the robot in a range of about 85% throttle, and only 11% extra thrust was obtained at the 100% throttle as shown in Fig. 16B. Without additional weight, it seems that free flight is possible under limited conditions with a small thrust deviation by control. However, if we consider mounting the vision system for missions, free flight becomes difficult.

### Attitude estimation

**Figure 17:**
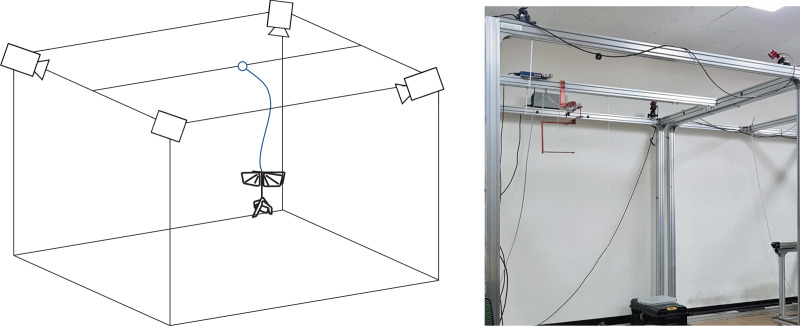
Attitude estimation experimental setup.

To evaluate the attitude estimation algorithm, a motion capture camera environment was established as shown in Fig. 17. The flight zone has a size of 2 × 1.5 × 2 m and, the motion capture camera system provides a quaternion-based attitude and position at 120 Hz. After attaching the five markers to the robot, a tethered flight test was performed. Due to the small number of cameras and limited Field Of View (FOV) of cameras, the marker intermittently disappeared from the camera’s FOV depending on the robot’s attitude, resulting in unwanted distortion such as impulse noise. To prevent the distortion and suppress noise, a Butterworth low filter of fourth-order with a 10 Hz cutoff frequency was applied to the reference value. Simultaneously, sensor measurements were stored at 500 Hz *via* the SD card module and then the attitude was estimated by the complementary filter and the Kalman filter, respectively. The crossover gain of the complementary filter was set ${K_p} = 0.1$ and the integral gain was set to ${K_i} = 0.002$. In the Kalman filter, the covariance of the gyroscope was set ${\sigma _p} = {\sigma _q} = {\sigma _r} = {0.5^\circ }/s$ and the covariance of measurements was set ${\sigma _\phi } = {\sigma _\theta } = {5^\circ }$. The detailed matrix used by the Kalman filter are as follows. The yaw angle can be estimated through the magnetometer, but algorithms don’t use it for measurements because the yaw angle was not taken into account in this study.

$\eqalign{& {\rm Q = }\left[ {\matrix{ {\sigma _p^2} & {\rm 0} & {\rm 0} \cr {\rm 0} & {\sigma _q^2} & {\rm 0} \cr {\rm 0} & {\rm 0} & {\sigma _r^2} \cr } } \right],\,\,\,\,\,\,\,R = \left[ \matrix{\sigma _\phi ^2 \hfill \cr \matrix{ {\rm 0} & {\sigma _\theta ^2} \cr } } \right],\,\,\,\,\Gamma = \left[ {\matrix{ 1 & {\sin \phi \tan \theta } & {\cos \phi \tan \theta } \cr 0 & {\cos \phi } & { - \sin \phi } \cr 0 & {\sin \phi /\cos \theta } & {\cos \phi /\cos \theta } \cr } } \right] \cr & {\rm H = }\left[ \matrix{\matrix{ 1 & 0 & 0 \cr } \cr \matrix{ 0 & 1 & 0 \cr } } \right]}$

**Figure 18:**
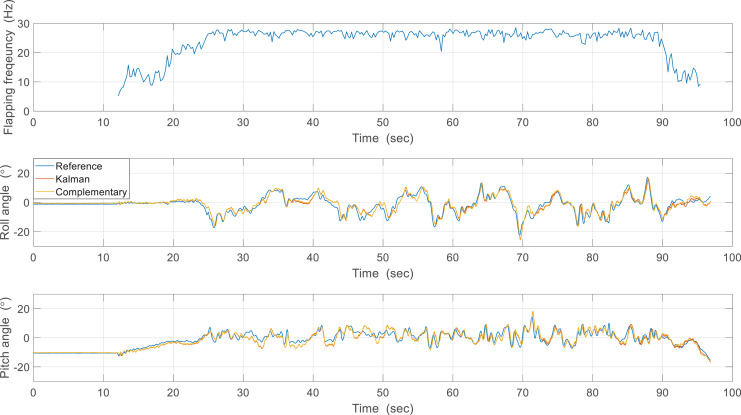
First estimation experiment results.

**Figure 19:**
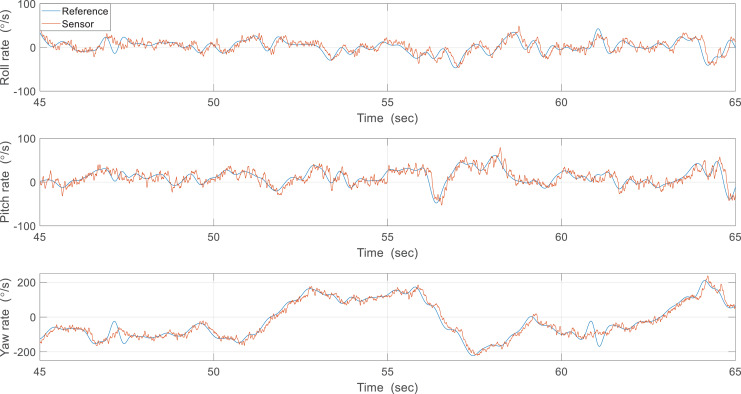
Reference angular velocities and the gyroscope measurements.

The first estimation experiment was carried out under the condition that the control surfaces of roll, pitch, and yaw were not used, and only the flapping frequency was given by manual input, which is shown in Fig. 18. The flapping frequency gradually increases from 0 s, reaches the maximum frequency at 25 s, and is maintained until 90 s. Figure 18 shows the attitude estimation results. The estimation error and time delay are very similar overall, and the performance was kept during experiments regardless of the flapping frequency. Table 2 shows the Root Mean Squared Error (RMSE) and time spent running the algorithm in Arduino with the robot processor for one interval. The RMSE of the Kalman filter is slightly smaller than that of the complementary filter, but the time running algorithm is about 90 times greater. This large execution time can degrade estimation performance by the increased discretization errors and can even adversely affect other real-time tasks. Therefore, the complementary filter was used as an onboard algorithm for all subsequent experiments. Furthermore, we compared the gyroscope measurements and the reference angular velocities obtained by differentiating the reference attitude. As shown in Fig. 19, the gyroscope measurements are delayed by filtering, but the robot’s rotational motion is captured well. This means that motion with a high flapping frequency has little effect on the estimation results through appropriate data filtering.

**Table 2:** Comparison of estimation errors and time running algorithms.

Filter	RMSE (roll/pitch)	Time
Kalman filter	2.12°, 2.15°	5.750 ms
Complementary filter	2.13°, 2.17°	0.065 ms

**Figure 20:**
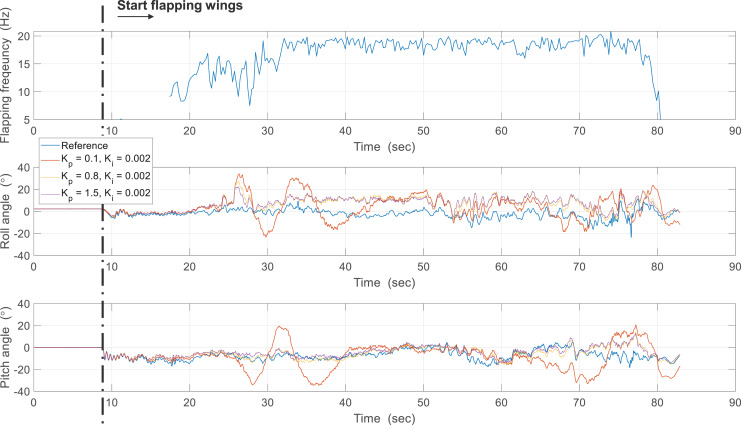
Results about effect of proportional gain.

**Figure 21:**
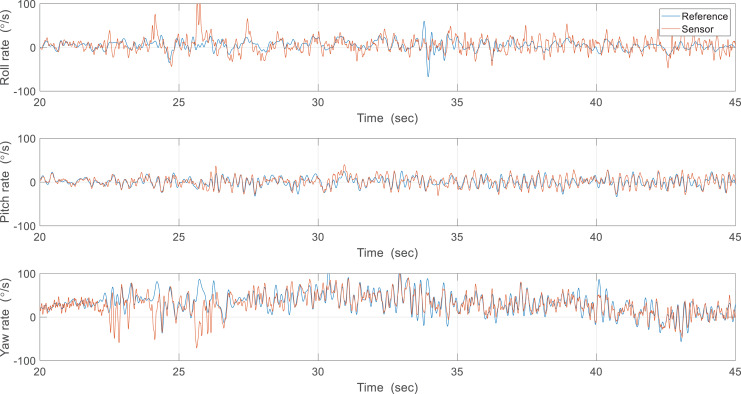
Reference angular velocities and the gyroscope measurements.

In the second experiment, the attitude was estimated in real-time using sampled sensor data at 200 Hz and a controlled flight experiment was conducted to hold the robot attitude. Figure 20 shows the attitude estimation results, and one can observe a clear estimation error after flapping wings. To identify the cause of errors, the reference angular velocities and the gyroscope measurements were compared from 20 to 45 s as shown in Fig. 21. While pitch rate measurements catch the actual rotational motion of the robot, the roll rate and yaw rate measurements do not do well, especially at around 25 and 34 s, and thereby result in considerable estimation errors. Unlike in the previous case, oscillations by flapping wings and moving control surfaces can be generated together in controlled flight. Since the robot is not exactly a rigid body and structural vibration occurs in the robot, the additional oscillation by control can distort the gyroscope measurements. To compensate for these errors, one can get help from the accelerometer measurements. Table 3 indicates estimation errors depending on the crossover band gain ${K_p}$. The pitch estimation error is significantly reduced, but the roll estimation error is not reduced for that time and this trend also can be found in Fig. 20. This results from the roll angle ${\phi _m}$ having a denominator only containing ${a_y}$ in Eq. (5), which is more sensitive to linear acceleration than the pitch angle having a denominator including ${a_y}$ and ${a_z}$. As a result, techniques to improve the signal-to-noise of the accelerometer or another aiding sensor that is not severely affected by oscillations are essential to get more accurate and robust estimation results.

**Table 3:** Comparison of estimation errors according to proportional gain of complimentary filter.

Gain set	${K_p} = 0.1$, ${K_i} = 0.002$	${K_p} = 0.8$, ${K_i} = 0.002$	${K_p} = 1.5$, ${K_i} = 0.002$
RMSE (roll/pitch)	10.69°, 11.27°	9.64°, 4.60°	9.84°, 4.55°

### Flight experiments

**Figure 22:**
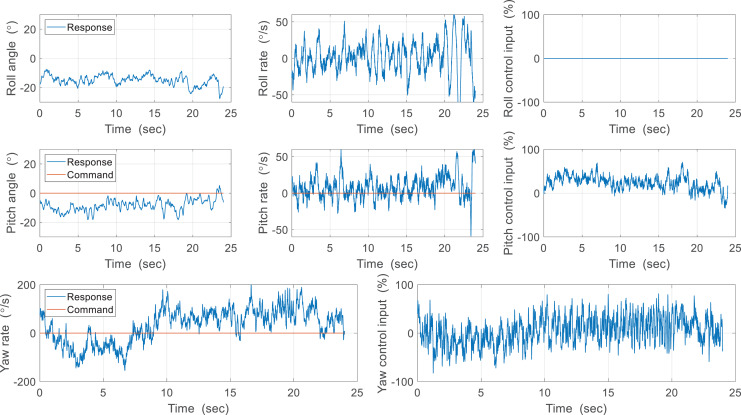
Free flight experiment results of the CUNX IV.

Here we deal with indoor free flight experiments of the CNUX IV and CNUX Mini. The CNUX IV has only two control surfaces of the elevator and the aileron for pitch and yaw control due to limited weight, so the roll control was not conducted. The controller architecture of the CNUX IV is slightly different from the aforementioned cascaded structure and consisted of one single loop. A command of 0° was applied to perform the hovering, and the flight experiment results of the CNUX IV are shown in Fig. 22. The roll was uncontrolled as there was no control surface, but the roll angle stabilized around −15° which appeared to be a trim roll angle. Alternatively, the value may not be the trim angle because the robot configuration is symmetric about the x-axis and z-axis of the center of gravity. Referring to the previous attitude estimation results, it can be the estimation error due to the linear acceleration by flapping wings. Control inputs were continuously generated for pitch and yaw, but responses didn’t regulate the control error. Pitch control error arises from not only the control effectiveness but also the small control gain. Whereas the pitch control performance can be slightly improved by increasing the control gain, the yaw control performance is hard to be enhanced since the yaw rate deviations range from −100 to +100 °/s despite the large deflection of the aileron. It can be confirmed that the robot can be stabilized by the tail, and yaw control is difficult for CNUX IV control mechanism. This is consistent with the control effectiveness problem mentioned earlier.

**Figure 23:**
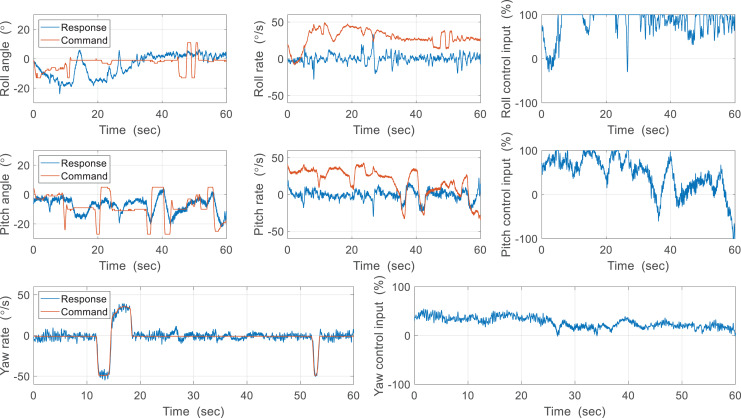
Free flight experiment results of the CUNX Mini.

**Figure 24:**
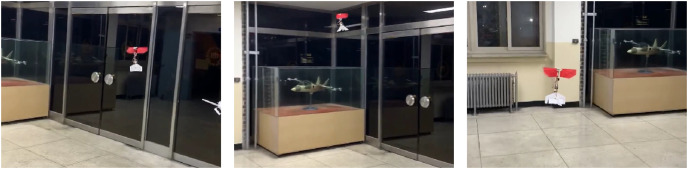
Photographs of flight experiment for the CUNX Mini.

**Figure 25:**
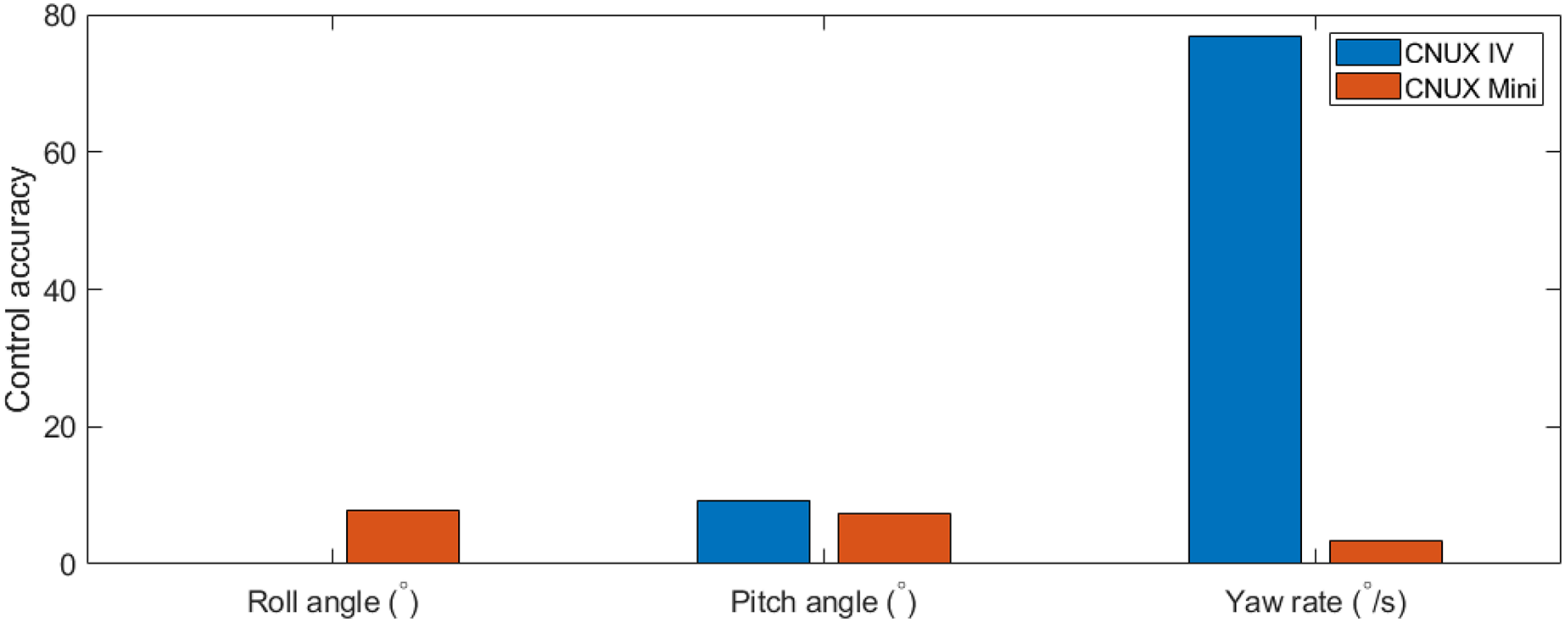
Comparison of control accuracy between CNUX IV and Mini.

The flight experiment of the CNUX Mini was performed indoors for 1.5 min and the results and photographs are represented in Figs. 23 and 24. To examine the control effectiveness of the control mechanism, we manually gave commands such as step input under hovering and slow forward flight conditions. Although the deflection area of the tail was increased, the roll rarely follows commands, and the pitch only responds to commands in a narrow range. Besides, there was a steady-state error even in the presence of integral control, and there was a considerable time delay. On the contrary, yaw followed commands very well over the flight time, especially at 10 to 20 s and around 53 s. There was no steady-state error through the integral control, and the time delay was not as large as the roll and pitch. Most of the yaw control inputs were used to compensate for steady-state errors, and much less control inputs were needed to follow the step input. This means that the new yaw mechanism can generate enough control moments and can handle a wide range of commands. The control accuracy of each axis for both robots is depicted in Fig. 25, and one can be aware of the considerable improvement in the yaw axis. Consequently, this experiment demonstrates that the new yaw control mechanism shows better control performance, but we still face the problem that the tail does not sufficiently provide the control force for roll and pitch. It seems that the control performance in roll and pitch did not improve significantly because the new yaw control mechanism eliminates the central section of the wing, reducing the effective area affected by wakes more than before.

Table 4 compares the overall flight performance of the robot with other X-type configuration-based flapping wing robots of similar size and weight. The thrust-to-wingspan ratio of the CNUX Mini increased by 32% compared to our previous work CNUX IV and outperformed even the NUS-Roboticbird and DelFly Nimble, which have the advantage of using two motors to obtain thrust. This means that not only the flapping mechanism was optimized, but also elements such as wing sizing, motor selection, and gear system reduction ratio were in balance. Nevertheless, the CNUX Mini requires a lot of energy to fly, and the flight time is reduced, which resulted from non-integrated avionics accounting for 19% of the total weight. In other words, if the weight of all systems is optimized, the flight time will increase, and the flight envelope can be extended from low to high speed.

**Table 4:** Comparison of flight performance with existing flapping wing robots.

Flapping wing robot	Mass (g)	Span (cm)	Thrust-to-wingspan ratio (N/m)	Flight time (min)
CNUX Mini	45	27	1.82	1.5
CNUX IV	37.1	29.4	1.37	6
DelFly Explorer	20	28	>=0.70	9
NUS-Roboticbird	31	22	1.59	3.5
DelFly Nimble	29	33	1.49	5

### Obstacle avoidance

**Figure 26:**
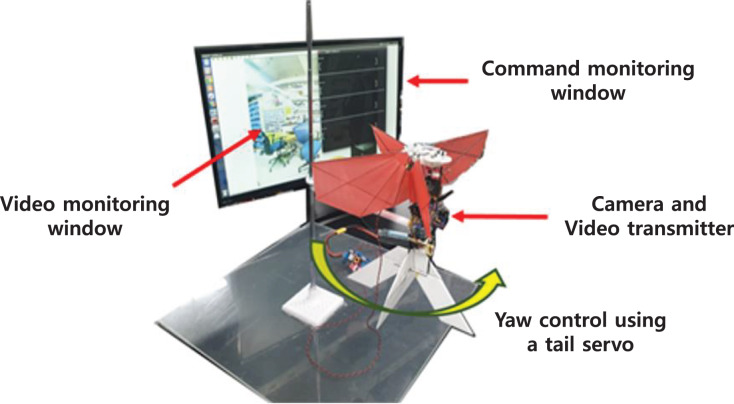
The testbed for obstacle avoidance algorithm.

The obstacle avoidance experiment was conducted on the testbed for counting a success rate, as shown in Fig. 26. Since the robot is fixed to the testbed, it is different from the actual flight maneuver due to constrained motion; this experiment is valid if the flapping wing robot does not significantly adjust pitch and roll during flight to maintain near hover conditions. Also, it can reflect the vibrations caused by the flapping of the wings during flight. The yaw rate command generated by the obstacle avoidance algorithm is realized *via* a tail servo motor.

**Figure 27:**
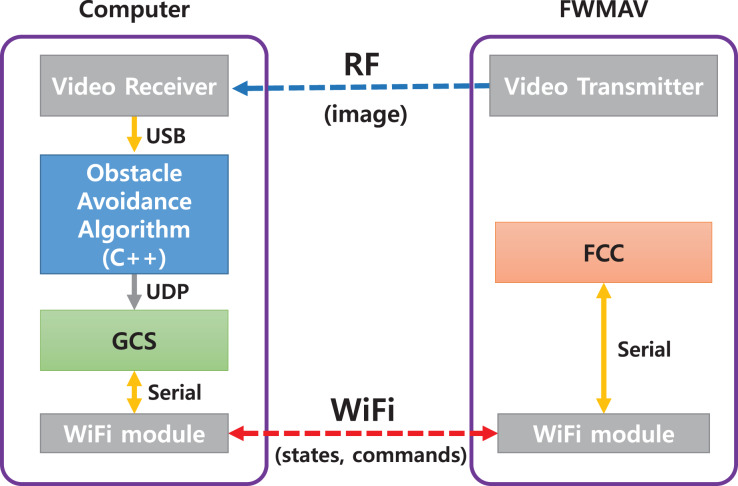
The structure of the obstacle avoidance system.

Figure 27 shows the structure of the obstacle avoidance system. The images are transmitted analogically through the wireless transmitter and receiver. The obstacle avoidance algorithm passes control commands to GCS through UDP communication. Finally, the GCS transmits the yaw rate command to the Flight Control Computer (FCC) through WiFi communication.

The robot is assumed to fly forward with a constant speed, and the obstacle was manually approached to the robot instead of an actual flight. The throttle input was set to $80\%$ of the maximum value to check the effect of the motion blur on the image. The approach speed of the obstacle was simulated with the general operating speed (0.5~1.0 m/s) of the robot. We tuned the obstacle avoidance threshold values ( ${\theta _{OF}},{\theta _H}$) so that the obstacle detector responds to the obstacle within 4 m considering the turning radius of the robot.

**Figure 28:**
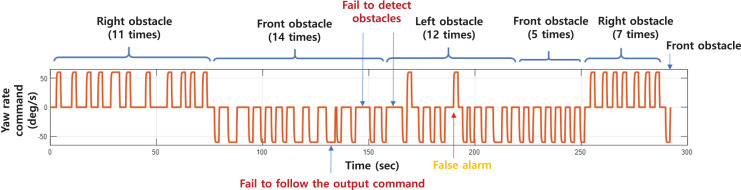
The yaw rate command generated during the experiment.

The experiment was conducted with 50 incoming obstacle situations. The yaw rate command generated by the obstacle avoidance algorithm is shown in Fig. 28. The time and direction of the incoming obstacle is indicated in the figure. An obstacle was not identified at 145 and 160 s, and a false alarm was generated even though there was no obstacle at 180 s. In addition, even though the control command was generated in 130 s, the robot did not respond, which seems to be due to poor communication or control. In most cases, incoming obstacles were correctly identified and avoided. The experimental results with obstacle avoidance success rate are shown in Table 5. The success of obstacle avoidance was determined by whether the robot performed the avoidance maneuver when an obstacle approached. The robot succeeded in avoiding 46 times out of a total of 51 times (including a false alarm), achieving a success rate of 90.2%.

**Table 5:** Performance of the obstacle avoidance algorithm.

	Left obstacle	Front obstacle	Right obstacle	Total
# of successful avoidances	10	18	18	46
# of obstacles approached	12	20	18	51
# of false alarms	1			

The proposed algorithm performs downsampling to reduce the effect of image noise, but fails to detect obstacles in the following situations. First, when the image is severely damaged due to radio wave interference, a false alarm is generated. Second, when the illuminance of the entire image is rapidly changed due to the shadow of an obstacle, there was a problem in recognizing side obstacles due to the limitation of the optical flow algorithm that is dependent on the intensity value of the image. This limitation can be improved through more detailed experimental setup, such as increasing the communication strength of the camera or canceling the auto exposure function so that the camera does not control the intensity depending on the total amount of light.

## Conclusions

In this article, we have systematically conducted research on the development of a flapping wing robot and an obstacle avoidance strategy that can be used in low-textured environments with low-quality cameras. We verified the flight performance of the flapping wing robot in the flight experiment and the obstacle algorithm in the ground experiment. The two experiments showed the potential for our robot to fly autonomously. However, overall performance evaluation should be done through flight experiments in an indoor environment with lots of obstacles. To achieve this, it is necessary to reduce the mass enough to fly with the vision system on board and to increase control moments to accurately follow commands on the roll and pitch axes. These issues are related to the redesign of the robot and will be addressed in further studies.

The proposed obstacle avoidance strategy uses light computational power; this makes it easy to adapt to miniaturized robots that require a lightweight CPU. In actual flight, when the robot flies forward in the middle of the corridor, the optical flow is symmetric, so the algorithm for detecting horizontal obstacles using the difference in the optical flow from the left and right sides is still effective. However, it is important to understand the effect of the pitch angle change of the robot during forward flight as it causes a significant change in the optical flow; this will be dealt with in future work. In addition, an obstacle avoidance method using a simple state machine was proposed when the robot encounters an obstacle on the ground testbed, but more rigorous algorithms could be required for real flight, which remains as future work.

## Additional Information and Declarations

### Data Availability

The following information was supplied regarding data availability: The data and code are available at GitHub: https://github.com/Heetae-Park/Flapping-wing-robot-project; Heetae-Park. (2022). Heetae-Park/Flapping-wing-robot-project: v1.0.0 (v1.0.0). Zenodo. https://doi.org/10.5281/zenodo.7418453.
